# Lactose intolerance awareness among adults in Riyadh, Saudi Arabia

**DOI:** 10.1016/j.heliyon.2024.e40272

**Published:** 2024-11-09

**Authors:** Nouran Aleyeidi, Latifah Albeheiri, Fatimah Alrashed, Abeer Alangari, Atha Alghazi, Jinan Alzaid, Nariman Sheikh, Renad Alqahtani, Sarah Alajmi, Tafe Albogami

**Affiliations:** College of Medicine, Princess Nourah bint Abdulrahman University, P.O. Box 84428, Riyadh, 11671, Saudi Arabia

**Keywords:** Lactose intolerance, Gastrointestinal diseases, Knowledge, Saudi Arabia

## Abstract

**Introduction:**

Lactose intolerance is a common medical condition characterized by malabsorption of lactose. Despite the considerable impact of lactose intolerance on affected persons, studies on its awareness are limited. The aim of study was to assess the awareness of lactose intolerance and its associated factors among the general population of Riyadh City, Saudi Arabia. The prevalence of lactose intolerance in the study population was also assessed.

**Methods:**

This was a cross-sectional study conducted in Saudi Arabia. An online questionnaire, which included 15 questions on lactose intolerance awareness and the risk factors for lactose intolerance, was administered. The prevalence of lactose intolerance was assessed using a validated five-question screening tool. The student's t-test and analysis of variance were used to compare quantitative variables, whereas the chi-square test was used to determine the association between qualitative variables.

**Results:**

A total of 2150 adult participants were included. The mean percentage of correct answers to questions regarding lactose intolerance awareness was 28.9 % (±21.9). Participants with a previous lactose intolerance diagnosis had the highest lactose intolerance knowledge scores (46.9 [±18.3]). Regarding the prevalence of lactose intolerance, 14.7 % of the participants responded that they were likely lactose intolerant, whereas 7.3 % reported that they were diagnosed with lactose intolerance by a physician. Results of the assessment performed using the screening tool, which had a sensitivity of 47 % in this study, showed that women were more likely to have lactose intolerance than men. In addition, women and younger participants had greater knowledge of lactose intolerance than men and older participants.

**Conclusion:**

The Saudi Arabian population has a low awareness of lactose intolerance. The findings of this study provide valuable information regarding level of lactose intolerance awareness among the Saudi population and can be used for the development of effective programs for increasing lactose intolerance awareness in Saudi Arabia.

## 1 Introduction

Lactose intolerance, or hypolactasia, is a common medical condition characterized by malabsorption of lactose in the small intestine caused by low levels of lactase, the enzyme that catalyzes the breakdown of lactose. This condition results in specific symptoms such as bloating, abdominal pain, diarrhea, nausea, and vomiting, which can be managed by switching to a lactose-free diet [1]. The main diagnostic test for lactose intolerance is the hydrogen breath test.

Three types of lactose intolerance have been identified so far. The first is congenital lactase deficiency, which is an extremely rare autosomal recessive disorder characterized by the absence or deficiency of lactase from birth. The second type is primary lactose intolerance, which is also an autosomal recessive disorder and the most common type of lactose intolerance. Primary lactose intolerance manifests in late childhood or adolescence. The third type of lactose intolerance is secondary lactose intolerance. Secondary lactose intolerance is not a genetic disorder and is characterized by transient lactose intolerance after intestinal damage caused by celiac disease, intestinal infections, Crohn's disease, ulcerative colitis, food allergies, or radiation/chemotherapy-induced enteritis [2].

Historically, the lactase persistence (LP) alleles spread in humans around 10,000 years ago as animal domestication began in the Old World, leading to the consistent inclusion of milk and dairy products in the diet. The spread of LP alleles varies today in different populations based on their history of dairy product consumption and of migration [3]. Nearly 20 years ago, genetic testing began to be more commonly used to evaluate LP and thus detect lactose malabsorption. However, the conventional tests for this purpose are the hydrogen breath and lactose tolerance tests; the gold standard test is endoscopic duodenal biopsy, although it is rarely used in clinical practice [4]. Lactose malabsorption is prevalent worldwide, with significant regional variations. A systematic review conducted in 2016 indicated that approximately two-thirds (68 %) of the global population have lactose intolerance. The highest prevalence was observed in Africa and Asia, excluding some countries like Saudi Arabia. The same study indicated that the prevalence of lactose intolerance in Saudi Arabia is 28 %. Recognizing the regional patterns of lactose malabsorption is important for managing gastrointestinal symptoms and increasing awareness of lactose intolerance in the general population [4]. This is particularly important because self-diagnosis of lactose intolerance based on symptoms is insufficient and may lead to an unwarranted avoidance of important dietary sources of calcium. Therefore, it is necessary to not only increase lactose intolerance awareness but to also educate the general population on the importance of confirming the causes of symptoms clinically and obtaining an accurate diagnosis from a physician [5].

Lactose intolerance is frequently mistaken for an allergy to cow milk by both patients and physicians. Cow milk allergy is an immune-mediated reaction to the protein in cow milk rather than an enzyme deficiency. Cow milk allergy peaks during the first year of life and tends to diminish before the age of five. The symptoms of cow milk allergy include skin rash, pruritus, angioedema, nausea, vomiting, and diarrhea; however, the non-IgE-mediated type is characterized by gastrointestinal symptoms such as vomiting, diarrhea, and blood in the stool, with or without mucus. Cow milk allergy is managed by consuming a diet without cow milk protein [2].

The awareness of lactose intolerance among physicians has been assessed in a few studies, whereas that among the general population has been investigated in even fewer studies. In a study on the awareness of lactose intolerance among the general population, which was conducted in India in 2020, 71.5 % of the 211 participants knew the term “lactose intolerance” and most of them acquired their knowledge of this term from the internet [6]. A similar study on lactose intolerance awareness in Saudi Arabia was published in 2022. In that study, data from 1189 participants were collected using an online questionnaire and those with a score of 60 % or higher were considered to have good awareness of lactose intolerance; only 38 % of the study population had good awareness of lactose intolerance [7].

Despite its high prevalence, studies on the awareness of lactose intolerance are limited. Awareness is the primary step in preventing complications associated with lactose intolerance, such as malnutrition and osteoporosis. Therefore, the aim of the present study was to evaluate the awareness of the risk factors for lactose intolerance and knowledge of its symptoms, complications, diagnosis, and management among adults in Saudi Arabia. In addition, we assessed the prevalence of lactose intolerance in the study population and analyzed the factors that affect the level of lactose intolerance awareness, including education, sex, age, employment in the healthcare field, and healthcare education.

## 2 Materials and methods

### 2.1 Study design, participants, and setting

This was a cross-sectional questionnaire-based study conducted in Riyadh City, the capital of Saudi Arabia, between December 15, 2021, and May 12, 2022. Adults living in Saudi Arabia, aged 18 years or older, were included.

### 2.2 Data collection

The participants completed an online self-administered questionnaire. They were asked to complete individual surveys through a survey link created using the REDCap electronic data capture tools [8]. The survey was sent through multiple online platforms such as email, WhatsApp, and Twitter. The questionnaire included items on sociodemographic data, including age, sex, nationality, educational level, history of healthcare education, and occupation. It also included 15 questions on the awareness of the causes, precipitating factors, symptoms, complications, diagnosis, and management of lactose intolerance. These questions were obtained from a validated questionnaire used in another study to assess lactose intolerance awareness among clinicians in Bahrain in 2018 [9]. However, four of the questions were modified and two were replaced. The questionnaire also included questions on the diagnosis of lactose intolerance and its risk factors.

A five-item, home-administered, and validated screening tool was used to identify participants with lactose intolerance [10]. The screening tool includes questions on common lactose intolerance symptoms such as diarrhea, stomachache, vomiting, noticeable abdominal sounds, gas, and meteorism. A score of seven points or higher (out of a possible total of 10 points) indicates the presence of lactose intolerance.

### 2.3 Sample size

The target sample size was calculated using the formula for calculating the sample size of a single proportion. We calculated the sample size based on the results of a previous study that revealed that the prevalence of lactose intolerance in Saudi Arabia was 28 % [4]. The sample size was calculated with a 95 % confidence level and a 5 % margin of error. We added 25 % more participants on the calculated sample size to compensate for any incomplete or missing data because this was an online questionnaire-based study. Therefore, a sample size of 1921 participants was required.

### 2.4 Sampling technique

The participants were recruited using convenience sampling.

### 2.5 Statistical analysis

Descriptive statistics are presented as means and standard deviations (SDs) for quantitative variables and frequencies and percentages for qualitative variables. Quantitative data were analyzed using t-tests or analysis of variance. The chi-square test was used to determine the association between qualitative variables. Statistical significance was set at P < 0.05. All statistical analyses were conducted using SPSS version 21.0 (IBM Corp., Armonk, NY, USA).

## 3 Results

A total of 2150 participants who fulfilled the inclusion criteria completed the questionnaire. The section on demographic characteristics included eight items (Table 1). The mean age of the participants was 36 years and 87.9 % of them were females. Most of the participants had a university degree and 86.8 % did not work in the healthcare field. Regarding nationality, 95 % of the participants were Saudis, whereas the other participants were of other nationalities. According to the latest Saudi census in 2022, the proportion of Saudis and non-Saudis in the population is 58.4 % and 41.6 % respectively. Also, 61.2 % of the participants were male and 38.8 % were female [11]. Since this study utilized a convenient non-probability sampling technique, a Chi-square goodness of fit test was applied to evaluate whether the study data were representative of the entire Saudi population with respect to sex and nationality. Regarding sex and nationality, the test results showed a significant difference between the study sample and the Saudi population (p < 0.001 for both), which implies that the sample was not representative of the entire Saudi population.

**Table 1:** Participant characteristics.

Variables	n (%)
**Age** (years)	36.4 ± 13.27^a^
**Sex**	
Female	1889 (87.9 %)
Male	261 (12.1 %)
**Nationality**	
Saudi	2042 (95 %)
Not Saudi	108 (5 %)
**Marital status**	
Married	1375 (64 %)
Unmarried	775 (36 %)
**Education**	
School	477 (22.2 %)
University degree	1462 (68 %)
Postgraduate degree	210 (9.8 %)
**Healthcare education (student/graduate)**	
Yes	283 (13.2 %)
**Occupation**	
Healthcare employee	77 (3.6 %)
Other type of employee	625 (29.1 %)
Unemployed	153 (7.1 %)
Retired	257 (12 %)
Homemaker	548 (25.5 %)
Student	490 (22.8 %)
**Family income**	
<10,000 SR	589 (27.4 %)
10,000–30,000 SR	965 (44.9 %)
>30,000 SR	246 (11.4 %)
No steady income	350 (16.3 %)
**Diagnosed with lactose intolerance**	
Yes	158 (7.3 %)
**Diagnosed with Crohn's disease**	
Yes	45 (2.4 %)
**Diagnosed with celiac disease**	
Yes	41 (2.1 %)
**Results of the lactose intolerance screening** (n = 1949) b	
No lactose intolerance	1663 (85.3 %)
Lactose intolerance	286 (14.7 %)

SR, Saudi riyal.

± standard deviation.

a Mean.

b The lactose intolerance screening was performed only for those who did not report a previous diagnosis of lactose intolerance.

The prevalence of lactose intolerance in the study population is presented in Table 1. Only 7.3 % of the participants reported that they were diagnosed with lactose intolerance by a physician. Regarding other diseases, 2.4 % of the participants were diagnosed with Crohn's disease and 2.1 % were diagnosed with celiac disease.

**Fig. 1:**
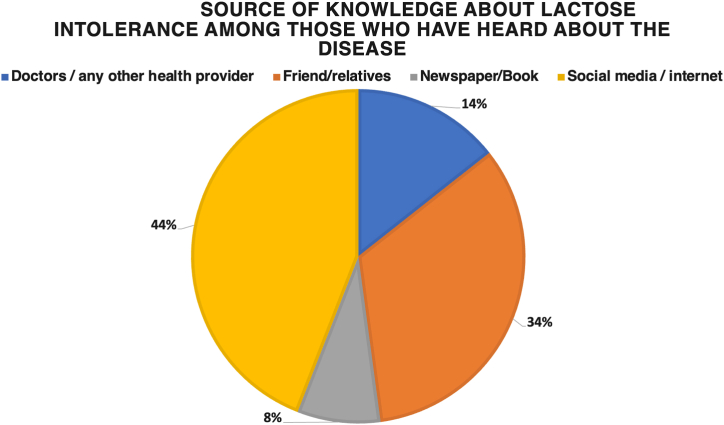
Source of knowledge about lactose intolerance among those who have heard about the disease.

Assessment performed using the five-item screening tool for lactose intolerance showed that 14.7 % of the participants who were not diagnosed with lactose intolerance were likely to have lactose intolerance. This group had greater knowledge of lactose intolerance than those who were not likely to have lactose intolerance. Regarding the participants’ knowledge of lactose intolerance, 30.2 % reported that they had never heard of lactose intolerance as a disease. Among those who had heard of lactose intolerance, most had learned about it through social media or other internet resources, 40.5 %. Approximately one-third of the participants stated that they had heard of it from a friend or relative. The least frequently mentioned source of knowledge was newspapers or books, 7.4 % (Fig. 1). Approximately half of the participants reported that they know someone with lactose intolerance, such as a family member, 22.5 %, or a friend, 17.8 %. The remaining participants, 59.7 %, did not know of anyone with lactose intolerance.

**Fig. 2:**
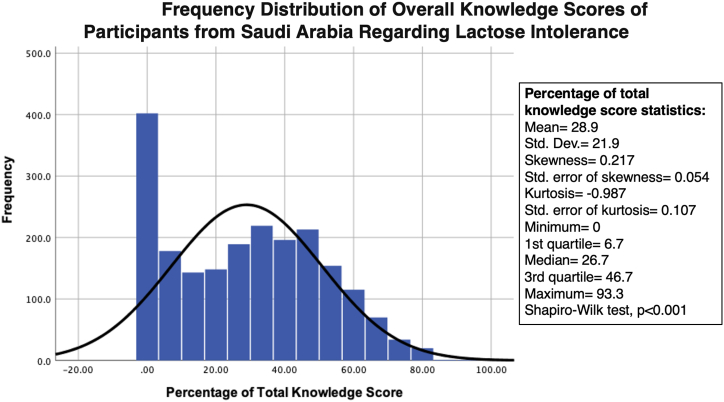
Frequency distribution of overall knowledge score of participants from Saudi Arabia regarding lactose intolerance.

The mean knowledge score regarding lactose intolerance was 28.9 %, with an SD of 21.9. The minimum score was zero and the maximum was 93.3 % (Fig. 2). The participants’ responses to the knowledge assessment items are presented in Table 2. The results indicated that 72.2 % of the participants recognized that lactose intolerance is not an infectious disease. This had the highest percentage of correct answers among all the questions on knowledge of lactose intolerance. The least correctly answered question was “Are lifelong management strategies needed for lactose intolerance?”, followed by “Can a child develop symptoms of lactose intolerance during the neonatal period?”; only 4 % and 8.6 % of the participants, respectively, provided correct answers to these questions. Furthermore, 36.5 % of the participants thought that rashes and shortness of breath were symptoms of lactose intolerance, whereas 53.1 % did not know if they were symptoms of lactose intolerance.

**Table 2:** Responses to questions on the knowledge of lactose intolerance.

Variables	n (%)
**Is there a difference between lactose intolerance and milk allergy?**	
Yes (correct)	497 (23.6 %)
**Is lactose intolerance genetically inherited by all affected individuals?**	
No (correct)	483 (22.9 %)
**Is lactose intolerance an infectious disease that can spread from an affected individual to a healthy individual?**	
No (correct)	1521 (72.2 %)
**Do some gastrointestinal diseases result in lactose intolerance?**	
Yes (correct)	662 (31.4 %)
**Do all patients with lactose intolerance develop symptoms?**	
No (correct)	552 (26.2 %)
**Do all patients with lactose intolerance develop symptoms after eating bread?**	
No (correct)	455 (21.6 %)
**Do patients with lactose intolerance usually develop symptoms after drinking milk?**	
Yes (correct)	1117 (53.1.%)
**Can a child develop symptoms of lactose intolerance during the neonatal period?**	
No (correct)	181 (8.6 %)
**Are rashes and shortness of breath symptoms of lactose intolerance?**	
No (correct)	220 (10.4 %)
**Is diarrhea a symptom of lactose intolerance?**	
Yes (correct)	931 (44.2 %)
**Is intestinal biopsy commonly used to diagnose lactose intolerance?**	
No (correct)	241 (11.5 %)
**Are lifelong management strategies necessary for lactose intolerance?**	
No (correct)	84 (4.0 %)
**Are there surgical treatments for patients with lactose intolerance?**	
No (correct)	704 (33.5 %)
**Should patients with lactose intolerance avoid milk derived from plants?**	
No (correct)	841 (40.0 %)
**Is malnutrition a possible complication experienced by patients with lactose intolerance?**	
Yes (correct)	616 (29.3 %)

The mean number of correct answers to the questions on awareness of lactose intolerance was 4.3/15 (SD ± 3.3), which is equivalent to 28.9 % (SD, ±21.9). Table 3 shows the mean knowledge scores, presented as percentages, for the different categories, along with the statistical significance of the differences between these categories. The average score of participants aged 30 years or younger was 34.0 % (SD, ±22.2); participants older than 30 years had a lower average score of 26.1 % (SD, ±21.2). The knowledge scores of participants who are healthcare workers and those who have a healthcare education were high (43.7 % [SD, ±20.7] and 44.0 % [SD, ±19.0], respectively). However, the highest scores were achieved by those who were previously diagnosed with lactose intolerance (46.9 %; SD, ±18.3).

**Table 3:** Comparison of lactose intolerance awareness scores.

Categories	Mean score, % (±SD)	P value∗
**Sex**		
Male	20.6 (±22.8)	<0.001
Female	30.1 (±21.5)	
**Age**		
Younger than 30 years	34.0 (±22.2)	<0.001
30 years or older	26.1 (±21.2)	
**Nationality**		
Saudi	28.8 (±21.8)	0.233
Not Saudi	31.4 (±23.4)	
**Marital status**		
Married	26.7 (±21.3)	<0.001
Unmarried	32.9 (±22.3)	
**Education**		
School	25.2 (±21.9)	<0.001
University degree	29.3 (±21.5)	
Postgraduate degree	34.8 (±22.9)	
**Healthcare education**		
Yes	43.7 (±20.7)	<0.001
No	26.7 (±21.2)	
**Occupation**		
Healthcare employee	44.0 (±19.0)	<0.001
Other type of employee	24.6 (±21.7)	
Unemployed	31.7 (±22.2)	
Retired	22.8 (±18.7)	
Homemaker	27.8 (±21.3)	
Student	35.6 (±21.9)	
**Family income**		
No steady income	27.2 (±21.4)	<0.001
<10,000 SR	26.8 (±21.8)	
Between 10,000 SR and 30,000 SR	29.3 (±21.9)	
>30,000 SR	34.9 (±21.4)	
**Previously diagnosed with lactose intolerance by a physician**		
Yes	46.9 (±18.3)	<0.001
No	27.4 (±21.5)	
**Diagnosed with Crohn's disease**		
Yes	25 (±21.2)	0.278
No	28 (±21.9)	
**Diagnosed with celiac disease**		
Yes	42 (±21.3)	<0.001
No	28 (±21.9)	
**Lactose intolerance screening tool result**		
Positive	40.4 (±21.3)	<0.001
Negative	27.6 (±21.5)	

SD, standard deviation; SR, Saudi riyal.

∗P-values were calculated using an independent *t*-test or analysis of variance depending on the number of categories. Statistical significance was set at P < 0.05.

The results of the assessment conducted using the validated lactose intolerance screening tool showed that 14.7 % of the participants who were not previously diagnosed with the disorder were likely to be lactose intolerant. Table 4 shows the screening results categorized according to sex, age, nationality, and income. They were compared using the chi-square test. More women than men screened positive for lactose intolerance. The percentage of individuals aged <30 years who screened positive for lactose intolerance was 4.6 % higher than that of individuals aged >30 years. Individuals with an income of more than 10,000 Saudi riyals were less likely to have lactose intolerance.

**Table 4:** Results of the assessment conducted using the lactose intolerance screening tool (number of participants = 1949).

Variables		Lactose intolerance screening tool results, n (%)		P value∗
		No lactose intolerance	Lactose intolerance	
Sex	Male	214 (90.3 %)	23 (9.7 %)	0.019
	Female	1449 (84.6 %)	263 (15.4 %)	
Age	Younger than 30 years	596 (82.4 %)	127 (17.6 %)	0.007
	30 years or older	1067 (87 %)	159 (13 %)	
Nationality	Saudi	1581 (85.7 %)	270 (14.6 %)	0.660
	Not Saudi	82 (83.7 %)	16 (16.3 %)	
Monthly income	<10,000 SR	449 (85.4 %)	77 (14.6 %)	1.000
	≥10,000 SR	1214 (85 %)	209 (14.7 %)	

SR, Saudi riyal.

∗P values were calculated using the chi-square test. Statistical significance was set at P < 0.05.

Table 5 shows the sensitivity and specificity of the screening tool used in this study. The screening tool had a sensitivity of 47 %.

**Table 5:** Specificity and sensitivity of the screening tool.

	With diagnosis	Without diagnosis	Total
Positive	70	216	286
Negative	78	1585	1663
Total	148	1801	1949

Specificity: 88 % (1585/1801); sensitivity: 47 % (70/148).

## 4 Discussion

Knowledge and awareness are key elements in the adoption of healthy behaviors and prevention of health complications. The source of the knowledge acquired is equally important. Approximately 70 % of the participants in the present study had heard of lactose intolerance, a percentage comparable to that reported in a study conducted in India (71.5 %) [6]. However, in a previous study conducted in Saudi Arabia, only 45.8 % of the participants knew about lactose intolerance [7]. It is difficult to compare the overall results on knowledge of lactose intolerance between the present study and the study conducted in India because knowledge scores or the percentage of participants with knowledge of lactose intolerance was not assessed in the Indian study [6]. In a previous study of the Saudi population, 38 % of the participants correctly answered 60 % or more of questions related to knowledge of lactose intolerance [7], whereas only 11.6 % correctly answered 60 % or more of the knowledge-related questions. This may have been because the questionnaires used in the two studies were different.

Most of the participants in the present study had general knowledge of lactose intolerance, knew that the symptoms were triggered by milk consumption, and knew that the disorder was not infectious. However, most of the participants believed that rashes and shortness of breath are symptoms of lactose intolerance. Additionally, only 8.6 % of the participants correctly responded that children do not develop lactose intolerance symptoms during the neonatal period. Regarding the question on the need for lifelong management strategies for individuals with lactose intolerance, only 4 % answered correctly that lifelong management was not necessary. This was considered the correct answer because secondary lactose intolerance, a type of lactose intolerance, is a temporary condition. Additionally, gradual adaptation to lactose may occur in some cases of primary lactose intolerance because some beneficial bacteria in the gut that secrete lactase slowly increase under certain conditions when lactose-containing foods are progressively introduced into the diet [12]. However, these situations are not commonly observed because lactose intolerance usually requires lifelong dietary restrictions. This may explain why most participants answered this question incorrectly.

A comparison of knowledge scores among the different subgroups of participants showed that female sex, younger age, unmarried status, higher education level, healthcare education, higher income, and diagnosis of lactose intolerance or celiac disease were associated with higher knowledge scores. The participants with healthcare education, those who were employed in the healthcare field, and those diagnosed with lactose intolerance or celiac disease had the highest knowledge scores. In a previous study conducted to assess the awareness of food allergies among pediatricians in Kuwait, the mean number of correctly answered questions was 22.2 out of 38 (58.4 %). In addition, the pediatricians’ knowledge scores were significantly associated with age (older pediatricians had higher overall scores) but not with sex [13]. In another study conducted to assess the knowledge of lactose intolerance among clinicians in Bahrain, more than 50 % of the participants correctly answered most of the questions; however, older clinicians had significantly higher scores than younger clinicians [10]. A study of the general population conducted in Saudi Arabia showed that females and younger participants had better scores than males and older participants [7].

In the present study, 7.3 % of the participants reported that they had previously been diagnosed with lactose intolerance. This percentage is close to that reported in a study conducted in Saudi Arabia in 2022 (8.7 %) [7]. The results of the lactose intolerance screening conducted in the present study showed that 14.7 % of the participants were probably lactose intolerant. Furthermore, the results indicated that both female and older participants were more likely to have this disorder than males and younger participants. Notably, sex and age are not known risk factors for lactose intolerance [14]. The abovementioned findings of the present study were based on the responses provided in the validated five-item lactose malabsorption questionnaire, which had a sensitivity of 47 % in the present study and sensitivity of 88 % in the original validation study [9]. The specificity calculated in the present study was excellent (88 %); however, the original study indicated that the specificity of the tool was only 35 % [9]. This may be because we classified participants as previously diagnosed with lactose intolerance based on their self-reported responses rather than on the results of a standard diagnostic test.

The main strength of the present study is that it provides comprehensive data on lactose intolerance awareness among adults in Saudi Arabia and has the largest sample size among similar studies. However, this study has some limitations that must be acknowledged. First, given that the number of studies on lactose intolerance awareness, particularly in Saudi Arabia, is limited, we had to reformulate the awareness questions used our survey because they were previously used to assess the lactose intolerance awareness of physicians. Second, despite the efforts of the research teams to simplify the medical terms included in the questionnaire, the disorder seemed unfamiliar to the Saudi population. One question about ulcerative colitis was removed from the analysis because it was misunderstood by the participants and elicited a very high number of incorrect responses. Third, the convenience sampling technique used in this study may have limited the generalizability of the results to the entire Saudi population. The chi-square goodness of fit test showed that the study sample was not representative of the entire Saudi population in terms of sex or nationality.

## 5 Conclusion

This study demonstrated that the Saudi Arabian population has a low level of knowledge regarding lactose intolerance. The findings this study can serve as a benchmark for the general knowledge of lactose intolerance in Saudi Arabia and can facilitate the development of effective awareness programs. However, it should be noted that the five-item lactose malabsorption questionnaire used in this study needs to be re-evaluated. In addition, further research is needed to determine the exact incidence of lactose intolerance in Saudi Arabia. Additionally, educational programs and screening research are necessary to increase awareness of lactose intolerance in the Saudi population.

## CRediT authorship contribution statement

**Nouran Aleyeidi:** Writing – review & editing, Validation, Supervision, Project administration, Methodology, Funding acquisition, Formal analysis. **Latifah Albeheiri:** Writing – original draft, Visualization, Methodology, Investigation, Data curation, Conceptualization. **Fatimah Alrashed:** Writing – original draft, Visualization, Methodology, Investigation, Data curation, Conceptualization. **Abeer Alangari:** Writing – original draft, Visualization, Methodology, Investigation, Data curation, Conceptualization. **Atha Alghazi:** Writing – original draft, Visualization, Methodology, Investigation, Data curation, Conceptualization. **Jinan Alzaid:** Writing – original draft, Visualization, Methodology, Investigation, Data curation, Conceptualization. **Nariman Sheikh:** Writing – original draft, Visualization, Methodology, Investigation, Data curation, Conceptualization. **Renad Alqahtani:** Writing – original draft, Visualization, Methodology, Investigation, Data curation, Conceptualization. **Sarah Alajmi:** Writing – original draft, Visualization, Methodology, Investigation, Data curation, Conceptualization. **Tafe Albogami:** Writing – original draft, Visualization, Methodology, Investigation, Data curation, Conceptualization.

## Ethical declaration

Ethical approval was obtained from the Princess Nourah bint Abdulrahman University Institutional Review Board (IRB) prior to data collection. An Exempt IRB approval was issued (IRB Log Number: 21-0475) on December 11, 2021, because this research project posed less than minimal risk to the participants. The participants voluntarily participated after reading a clear description of the study. They had complete rights to refuse participation without any consequences. Written informed consent was obtained online from all participants before completion of the questionnaire. Confidentiality and anonymity of the participants was ensured.

## Data availability statement

The data associated with this study have not been deposited into a publicly available repository; however, the data will be made available on request.

## Funding

The authors extend their appreciation to the Deputyship for Research & Innovation, 10.13039/501100011821Ministry of Education in Saudi Arabia for funding this research work through the project number RI-44-0814.

## Declaration of competing interest

The authors declare that they have no known competing financial interests or personal relationships that could have appeared to influence the work reported in this paper.
